# Protein kinase C inhibition attenuates vascular ET_B _receptor upregulation and decreases brain damage after cerebral ischemia in rat

**DOI:** 10.1186/1471-2202-8-7

**Published:** 2007-01-09

**Authors:** Marie Henriksson, Emelie Stenman, Petter Vikman, Lars Edvinsson

**Affiliations:** 1 Division of Experimental Vascular Research, Department of Clinical Sciences in Lund, Lund University, Sweden

## Abstract

**Background:**

Protein kinase C (PKC) is known to be involved in the pathophysiology of experimental cerebral ischemia. We have previously shown that after transient middle cerebral artery occlusion, there is an upregulation of endothelin receptors in the ipsilateral middle cerebral artery. The present study aimed to examine the effect of the PKC inhibitor Ro-32-0432 on endothelin receptor upregulation, infarct volume and neurology outcome after middle cerebral artery occlusion in rat.

**Results:**

At 24 hours after transient middle cerebral artery occlusion (MCAO), the contractile endothelin B receptor mediated response and the endothelin B receptor protein expression were upregulated in the ipsilateral but not the contralateral middle cerebral artery. In Ro-32-0432 treated rats, the upregulated endothelin receptor response was attenuated. Furthermore, Ro-32-0432 treatment decreased the ischemic brain damage significantly and improved neurological scores. Immunohistochemistry showed fainter staining of endothelin B receptor protein in the smooth muscle cells of the ipsilateral middle cerebral artery of Ro-32-0432 treated rats compared to control.

**Conclusion:**

The results suggest that treatment with Ro-32-0432 in ischemic stroke decreases the ischemic infarction area, neurological symptoms and associated endothelin B receptor upregulation. This provides a new perspective on possible mechanisms of actions of PKC inhibition in cerebral ischemia.

## Background

Protein kinase C (PKC) was discovered almost thirty years ago by Takai et al [1]. It has since been shown to include several isoforms, all of which are serine/threonine kinases [2-4]. The PKC isoforms are divided into three subgroups; the conventional, the novel and the atypical PKCs depending on their structure and requirements for activation [3,5,6]. PKC is activated by a range of different stimuli, including growth factors and hormones [5], and it plays a key role in several cardiovascular diseases such as stroke and heart failure [7-9].

In previous studies, we have demonstrated that in experimental ischemic stroke and subarachnoid hemorrhage (SAH) there is an upregulation of endothelin type B (ET_B_) receptors in the cerebral arteries [10,11]. There are two known endothelin receptors in the vasculature of mammals, the endothelin A (ET_A_) and endothelin B (ET_B_) receptor [12]. The ET_B _receptors are normally situated on the endothelial cells, mediating dilatation, but in the case of SAH and experimental ischemic stroke there is an upregulation of contractile ET_B _receptors in the vascular smooth muscle cells [10,11]. This alteration is also seen in organ culture of middle cerebral arteries (MCA) [13]. In both SAH and organ culture this upregulation is attenuated by PKC inhibitors [13-15].

The aim of the present study was to examine if a general PKC inhibitor, Ro-32-0432, can decrease the ET_B _receptor upregulation in MCA and reduce the ischemic infarct volume after experimental ischemic stroke. Transient middle cerebral artery occlusion (MCAO) was induced by an intraluminal filament technique and Ro-32-0432 was injected intraperitoneally (i.p.) in conjunction with the occlusion. The Ro-32-0432 treatment decreased the ET_B _receptor upregulation, as well as diminished the ischemic infarct area and improved the neurological status of the animals. Moreover, immunohistochemistry showed enhanced expression of ET_B _receptor protein in the ipsilateral MCA of the control rats. This enhancement was not seen in the ipsilateral MCA of the Ro-32-0432 treated animals, which confirms the contractile results.

## Results

### Middle cerebral artery occlusion

In all included animals, a proper occlusion and reperfusion was confirmed by laser-Doppler flowmetry in the cortex area supplied by the MCA. There was no difference in blood flow between the control group and the Ro-32-0432 treated group. Before occlusion MAP, pO_2_, pCO_2_, pH and plasma glucose were measured, and there were no differences between the groups. The body temperature usually increases temporarily the first hours after MCA occlusion [16], a phenomenon confirmed in this study. There was no difference in weight loss during the reperfusion period. All physiological parameters in conjunction with the operation are summarized in Table 1.

The neurological scores differed between the groups; 3.83 ± 0.17 in control group compared to 3.00 in the Ro-32-0432 treated group (P < 0.05; n = 6 in control group, n = 4 in Ro-32-0432 treated group). For definition of the neurological scoring system, see Table 2.

### Infarct volume evaluation

**Figure 1:**
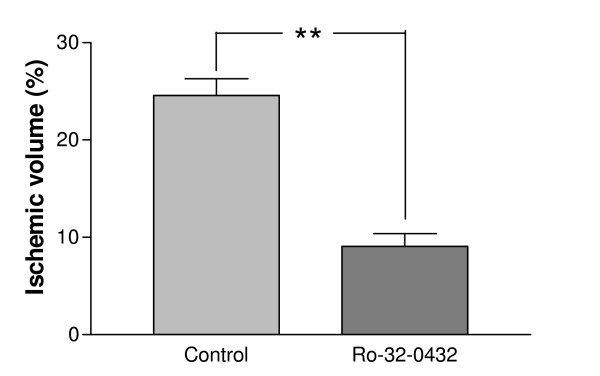
Ischemic infarction volume (% of total brain volume) in rats after MCAO in conjuction with treatment with vehicle (control) or Ro-32-0432. The PKC inhibition resulted in significantly smaller infarction volumes. Values represent means % ± S.E.M %, n = 4–6 (**P < 0.01).

Analysis of the infarct volume after staining with TTC revealed that treatment with Ro-32-0432 significantly decreased the size of the ischemic infarct area as compared to the control group; control: 24.6% ± 1.7% and Ro-32-0432: 9.1% ± 1.3% (Figure 1, **P < 0.01).

### Myograph experiments

K^+^-induced contractions did not differ between the control group and the Ro-32-0432-treated group (1,77 ± 0,18 mN compared to 1,85 ± 0,27 mN).

**Figure 2:**
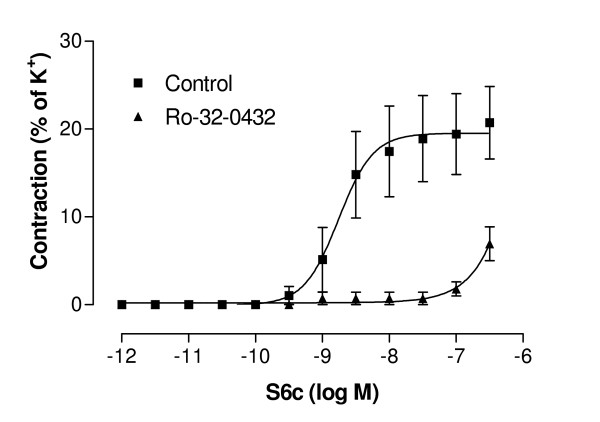
Contractile responses to S6c in the RMCA of vehicle or Ro-32-0432 treated rats after MCAO. The ischemia resulted in S6c induced contractile responses due to ET_B _receptor upregulation, which Ro-32-0432 was able to attenuate. Values represent means ± S.E.M., n = 4–6 (for details see Table 3).

The contractile response towards sarafotoxin 6c (S6c; selective ET_B _receptor agonist) was decreased in the right MCA of the Ro-32-0432 treated rats compared to the right MCA of the control rats 24 hours after the occlusion. This difference was significant at S6c concentrations of 10^-8.5 ^– 10^-6.5 ^M (Figure 2, *P < 0.05). Following S6c administration, the ET_B _receptors are desensitized, leaving only ET_A _receptors to interact with endothelin-1 (ET-1; ET_A_/ET_B _agonist) [17].

The contractile response towards ET-1 did not differ between the groups. However, there was a significant increase in the right MCA compared to the left MCA of the Ro-32-0432 group. All values are summarized in Table 3.

### Immunohistochemistry

**Figure 3:**
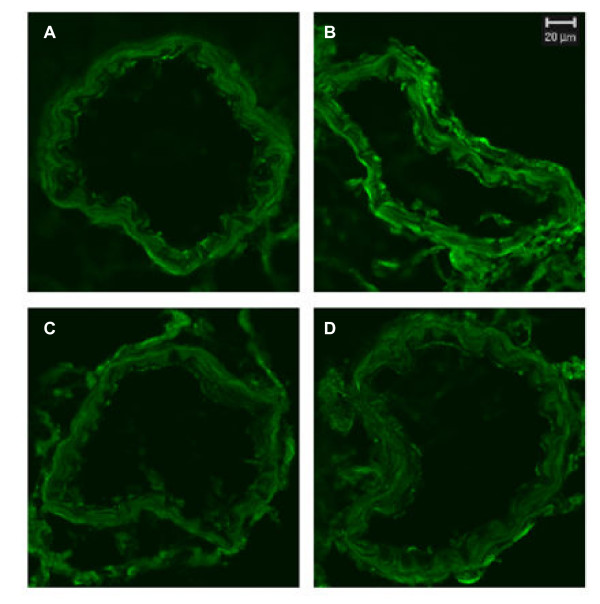
ET_B _receptor protein in (A) Ro-32-0432 RMCA, (B) control RMCA, (C) Ro-32-0432 LMCA and (D) control LMCA. There was an enhanced expression of ET_B _receptor protein in the smooth muscle cells in the ischemic RMCA (B). Treatment with Ro-32-0432 abolished this (A). Pictures were taken at 40× magnification.

In the control group, immunohistochemistry confirmed an enhanced expression of ET_B _receptor protein in the smooth muscle cells of the right MCA after transient MCAO. This enhancement was abolished in the right MCAs of the rats treated with the PKC inhibitor Ro-32-0432. There were no differences in ET_B _receptor staining between the left MCAs. n = 3 in all groups, figures are representative for the groups (Figure 3).

## Discussion

Protein kinase C has long been known to be involved in the pathophysiology of cerebral ischemia [18]. However, the underlying mechanisms of its involvement are still unclear, possibly due to different roles of the PKC isoforms in the pathophysiology of the disease [9]. For example, PKCδ activation in experimental cerebral ischemia has proven to be deleterious [19-21], while the PKCγ isoform is potentially beneficial [22].

Here we show for the first time that i.p. administration of Ro-32-0432, an inhibitor known for its PKC selectivity, decreases the infarct volume and improves the neurological score of the rats 24 hours after transient MCAO. Furthermore, the contractile ET_B _receptor upregulation seen in the ipsilateral MCA of the control animals is attenuated by the Ro-32-0432 treatment. It has previously been shown that there is an enhanced contraction of the pial vessels overlying the penumbra, which may worsen the ischemic damage [23]. A normalization of the vascular responses towards endothelin may contribute to the beneficial outcome of treatment with Ro-32-0432 in the present animal model of transient MCAO.

The effect of endothelin receptor inhibition in cerebral ischemia has been widely studied, but the results have not been conclusive [24-26]. Our group has previously shown that in addition to an ischemia-induced endothelin receptor upregulation, there is an alteration in the vascular angiotensin II receptor response after experimental cerebral ischemia [27]. This points towards a more general receptor modification in the affected arteries. Therefore a specific ET receptor blocker may not be the most useful way of treatment, but rather an inhibitor of the pathways leading to the receptor changes.

We have previously shown that inhibition of the (MAPK) extracellular-regulated kinase 1/2 (ERK1/2) pathway has a similar beneficial effect on ET receptor alterations, ischemic infarction area and neurological score after MCAO as the PKC inhibition in the present study [28]. This is also seen in organ culture, where the upregulation of contractile ET_B _receptors in MCA is attenuated by both PKC inhibitors and ERK1/2 inhibitors [13,14,29].

Since the ERK1/2 kinase can be phosphorylated and activated by PKC, this suggests a common intracellular pathway for these kinases in the cerebral ischemia pathophysiology. However, further studies are needed to confirm this connection and to elucidate which of the isoforms of PKC that are involved. Moreover, whether the decrease in cerebral infarct volume is due to PKC inhibition in the neuronal tissue or an effect of the attenuation of the ET_B _receptor upregulation in the arteries remains to be investigated.

## Conclusion

Treatment with the PKC inhibitor Ro-32-0432 reduces the upregulation of ET_B _receptors in the ipsilateral MCA 24 hours after transient middle cerebral artery occlusion. In addition, the ischemic infarction area is decreased and the neurological status improved by the PKC inhibition. These results provide new insights into the beneficial effects of PKC inhibition in cerebral ischemia.

## Methods

### Middle cerebral artery occlusion

Male Wistar Hannover rats weighing 350–400 g were obtained from Harlan, Horst, the Netherlands. The animals were housed under controlled temperature and humidity conditions with free access to tap water and food. The experimental procedures were approved by the University Animal Ethics Committee (M131-03). MCAO was induced by an intraluminal filament technique, previously described by Memezawa and colleagues [30]. Briefly, anesthesia was induced using 4.5% halothane in N_2_O:O_2 _(70%:30%). The rats were kept anesthetized by inhalation of 1.5% halothane through a mask. A filament was inserted into the right common carotid artery and further advanced through the internal carotid artery until it occluded the right MCA. To confirm a proper occlusion and subsequently a proper reperfusion of the right MCA, a laser-Doppler probe (Perimed, Sweden) was fixed on the skull (1 mm posterior to the bregma and 6 mm from the midline on the right side), measuring the blood flow in an area supplied by the right MCA. A polyethylene catheter was inserted into a tail artery for measurements of mean arterial blood pressure (MAP), pH, pO_2_, pCO_2 _and plasma glucose. A rectal temperature probe connected to a homeothermal blanket was inserted for maintenance of a body temperature at 37°C during the operation. Thereafter, an incision was made in the midline of the neck and the right common, external and internal carotid arteries were exposed. The common and external carotid arteries were permanently ligated with sutures. A filament was inserted into the internal carotid artery via an incision in the common carotid artery, and further advanced until the rounded tip reached the entrance of the right MCA. The resulting occlusion was made visible by laser-Doppler flowmetry as an abrupt reduction of cerebral blood flow with 75–90%. Immediately after occlusion, the rats were injected i.p. with either 30 mg/kg Ro-32-0432 dissolved in 0.6 ml dimethyl-sulfoxide (DMSO) or just 0.6 ml DMSO (control). The rats were then allowed to wake up.

Two hours after occlusion the rats were reanesthetized to allow for withdrawal of the filament and achieve reperfusion. Inclusion criteria were a proper occlusion (> 75% reduction of regional blood flow) as measured by laser-Doppler flowmetry. Rectal temperature was measured 30 minutes before occlusion and 1 hour after reperfusion. The rats were examined neurologically immediately before they were sacrificed, 24 hours after MCAO, according to an established scoring system (Table 2) [31-33].

### Infarction volume evaluation

The brains were sliced coronally in 2 mm thick slices, and stained with 1% 2, 3, 5-triphenyltetrazolium chloride (TTC) dissolved in physiological saline solution. The size of the ischemic infarction volume was calculated using the software program Brain Damage Calculator 1.1 (MB Teknikkonsult, Lund, Sweden). The swelling of the ischemic hemisphere was approximated by the ratio of the areas of the two hemispheres in the same slice. The infarction area values are compensated for this swelling before being used in the volume calculations. The infarction volume is calculated by numerical integration of the ischemic area of each slice using the trapezium rule and is expressed as percentage of total brain volume in the slices.

### Myograph experiments

Mulvany-Halpern myographs (Danish Myo Technology A/S, Denmark) were used for measurements of the arterial contractile properties [34,35]. The arteries were cut into cylindrical segments and the endothelium was removed mechanically by rubbing it off with a thread. The segments were mounted on two 40 μm diameter stainless steel wires and placed in the myographs. One of the wires was connected to a force transducer attached to an analogue-digital converter unit (ADInstruments, UK). The other wire was attached to a movable displacement device allowing adjustments of vascular tension by varying the distance between the wires. The experiments were recorded on a computer by use of the software program Chart™ (ADInstruments). The segments were immersed in a temperature-controlled (37°C) bicarbonate buffer of the following composition (mM): NaCl 119; NaHCO_3_15; KCl 4.6; MgCl_2 _1.2; NaH_2_PO_4 _1.2; CaCl_2 _1.5 and glucose 5.5. The buffer was continuously gassed with 5% CO_2 _in O_2_, resulting in a pH of 7.4. The arteries were given an initial tension of 1.2 mN, and were allowed to adjust to this level of tension for 1 hour. The contractile capacity was determined by exposure to a potassium-rich (63.5 mM) buffer with the same composition as the bicarbonate buffer solution except that NaCl was partly exchanged for KCl. Dose-response curves for S6c and ET-1 were obtained by cumulative application (10^-12^-10^-6.5 ^M). The E_max _values represent the maximum vascular contraction as response to S6c or ET-1 and were calculated as percentage of the contractile response towards 63.5 mM K^+^. The pEC_50 _values represent the negative logarithm of the concentration which elicits half-maximum response. A mean value of the segments of each MCA was calculated.

### Immunohistochemistry

The MCAs were placed onto Tissue TEK (Gibco, UK), frozen and subsequently sectioned into 10 μm thick slices in a calibrated Microm HM500M cryostat (Microm, Germany). The primary antibody used was polyclonal rabbit antirat, diluted 1:100 (AbCam). The secondary antibody used were donkey antirabbit Cy™3 conjugated (JacksonImmunoResearch, 711-165-152) 1:100, diluted in PBS with 10% fetal calf serum. The antibody was then detected at the appropriate wavelength in a confocal microscopy (Zeiss, USA). Pictures were taken at 40× magnification. As control, only secondary antibody was used.

### Calculations and statistical analyses

All data are expressed as mean values ± S.E.M. n = number of rats. Statistical analyses were performed with a non-parametric Mann-Whitney test. P < 0.05 was considered significant.

## Authors' contributions

**MH **participated in the design of the study, analyzed the ischemic infarction volumes, performed the myograph experiments and the statistical analysis and wrote most of the manuscript.

**ES **participated in the design of the study, analyzed the ischemic infarction volumes and revised the manuscript.

**PV **carried out the immunohistochemistry.

**LE **participated in the design of the study and revised the manuscript.

All authors read and approved the manuscript.

## Figures and Tables

**Table 1:** Physiological parameters during and after MCAO.

Physiological parameters	Control (n = 6)	Ro-32-0432 (n = 4)
pO_2 _(mmHg)	97.2 ± 7.1	91.1 ± 11.5
pCO_2 _(mmHg)	64.33 ± 4.6	62.2 ± 9.1
pH	7.309 ± 0.032	7.369 ± 0.03
Plasma glucose (mM)	9.78 ± 1.4	10.55 ± 0.7
MAP (mmHg)	98.7 ± 5.3	103 ± 2.5
Temperature during operation (°C)	37.1 ± 0.1	37.1 ± 0.1
Temperature after operation (°C)	38.9 ± 0.2	38.4 ± 0.3
Weight loss (%)	9.9 ± 4.6	9.9 ± 1.6

P > 0.05 between the groups.

**Table 2:** Neurological scores after MCAO.

Score	Interpretation
0	No visible deficits
1	Contralateral forelimb flexion, when hold by tail
2	Decreased grip of contralateral forelimb
3	Spontaneous movement in all directions, but contralateral circling if pulled by tail
4	Spontaneous contralateral circling
5	Death

**Table 3:** Contractile properties of the MCAs.

	**Control**				**Ro-32-0432**			
	
	RMCA		LMCA		RMCA		LMCA	
	
	E_max_(%)	pEC_50_	E_max_(%)	pEC_50_	E_max_(%)	pEC_50_	E_max_(%)	pEC_50_
**S6c**	21 ± 4	8.38 ± 0.30	3 ± 2**	6.81 ± 0.01*	7 ± 2*	7.43 ± 0.59	5 ± 3**	6.89 ± 0.16
**ET-1**	238 ± 19	7.62 ± 0.14	253 ± 28	7.49 ± 0.13	278 ± 11	7.54 ± 0.09	187 ± 12^†^	7.72 ± 0.31

Contractile responses to sarafotoxin 6c (S6c) and endothelin-1 (ET-1) in the right and the left MCA 24 hours after MCAO. E_max _is expressed as percent of K^+ ^induced contractions and pEC_50 _values represent negative logarithm of the agonist concentration producing half maximum response. *P < 0.05, **P < 0.01 compared to control RMCA. ^†^P < 0.05 compared to Ro-32-0432 RMCA.
